# Supplementary material for: Gut microbiota and age shape susceptibility to clostridial enteritis in lorikeets under human care
Source: Anim Microbiome. 2022 Jan 9;4:7. doi: 10.1186/s42523-021-00148-7 (PMC8744333; doi:10.1186/s42523-021-00148-7)
Supplement: Supplementary file 1 — Additional file 1. Experimental design. Lorikeet sampling by season and opportunistically during cases of enteritis at the a) Columbus Zoo and Aquarium and the b) Denver Zoo. A single cloacal swab was collected from each bird during flock surveys. In birds with enteritis, a single cloacal swab was generally collected at first presentation of illness. In a few birds, additional swabs or samples were collected over the duration of the illness or at necropsy (post-mortem samples). [file 42523_2021_148_MOESM1_ESM.pdf]

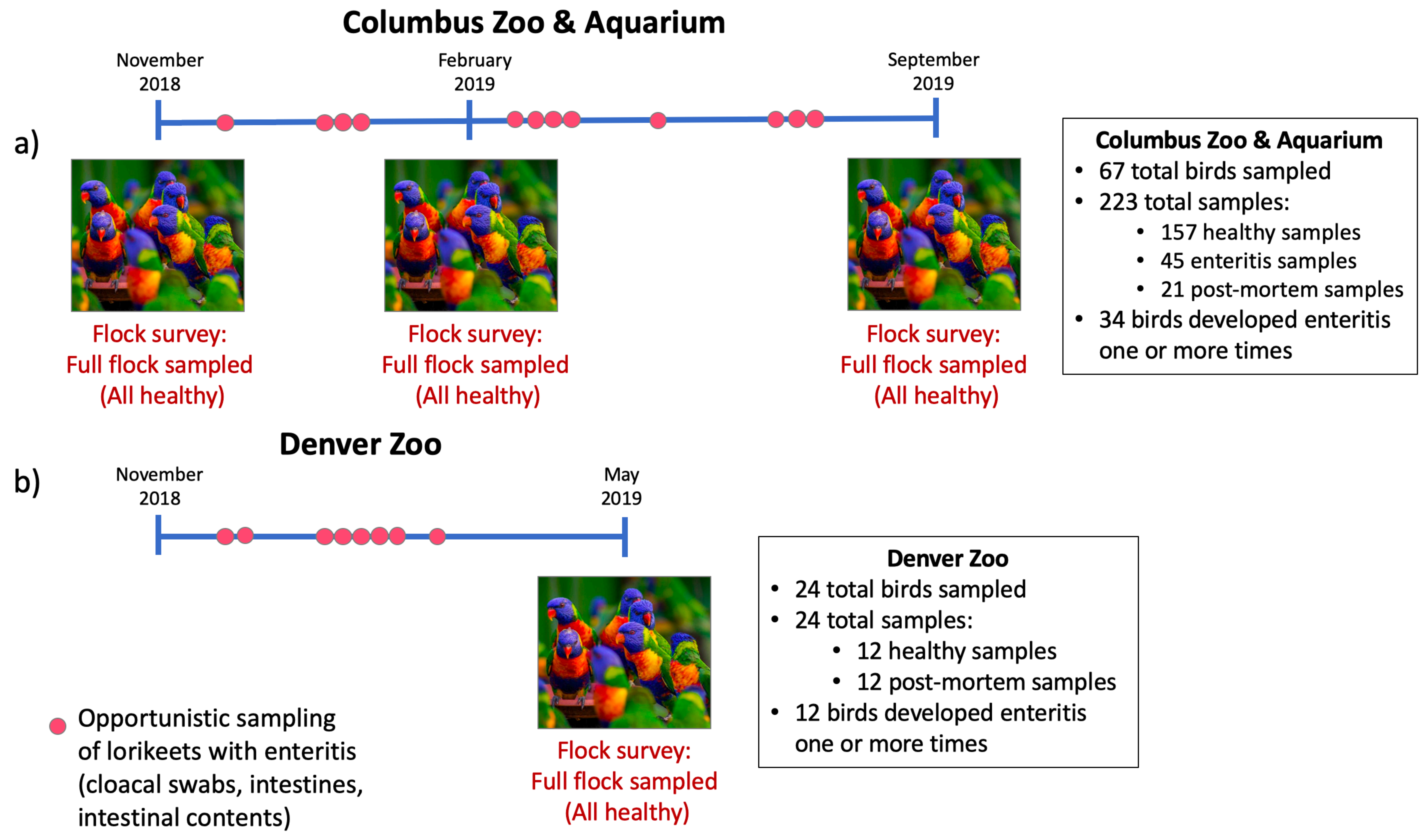

Columbus Zoo & Aquarium
a)
November 2018
February 2019
September 2019
Flock survey: Full flock sampled (All healthy)
Flock survey: Full flock sampled (All healthy)
Flock survey: Full flock sampled (All healthy)
Columbus Zoo & Aquarium
• 67 total birds sampled
• 223 total samples:
• 157 healthy samples
• 45 enteritis samples
• 21 post-mortem samples
• 34 birds developed enteritis one or more times
Denver Zoo
b)
November 2018
May 2019
Flock survey: Full flock sampled (All healthy)
Denver Zoo
• 24 total birds sampled
• 24 total samples:
• 12 healthy samples
• 12 post-mortem samples
• 12 birds developed enteritis one or more times
Opportunistic sampling of lorikeets with enteritis (cloacal swabs, intestines, intestinal contents)
