# Supplementary material for: Gut microbiota and age shape susceptibility to clostridial enteritis in lorikeets under human care
Source: Anim Microbiome. 2022 Jan 9;4:7. doi: 10.1186/s42523-021-00148-7 (PMC8744333; doi:10.1186/s42523-021-00148-7)
Supplement: Supplementary file 2 — Additional file 2. Clostridium perfringens IHC protocol from California Animal Health & Food Safety Laboratory. [file 42523_2021_148_MOESM2_ESM.pdf]

PURPOSE

To detect Clostridium perfringens in paraffin-embedded tissue sections.

SCOPE

Clostridium perfringens antibodies are labeled with a peroxidase labeled polymer and visualized with a colored substrate-product reaction.

DEFINITIONS AND ACRONYMS

1. IHC: Immunohistochemistry
2. TBS: Tris buffered saline

SPECIMEN INFORMATION

Formalin fixed, paraffin-embedded tissues.

REAGENTS AND MEDIA

1. Antigen Retrieval Solution
   Distilled Water ---150 ml
   Pepsin --- .6 gm
   Hydrochloric acid -- 1.5 ml
2. Endogenous Peroxidase Quenching Solution, 3%
   Distilled Water --135 ml
   Hydrogen peroxide (30%) -- 15 ml
3. Rinse Buffer pH 7.4
   TBS Autowash Buffer-20x(or equivalent)-------------------50ml
   Commercial Product-BioCare Medical
   Distilled Water -- 950 ml
4. Antibody Diluent
   DaVinci Green Diluent-or equivalent----------------------RTU
5. Blocking Solution
   Background Punisher or equivalent ---------------------------RTU
   Commercial Product-BioCare Medical
6. Primary Antibody Solution
7. Secondary Detection Solution
   Anti-rabbit polymer labeled with HRP or equivalent--------RTU
8. Nova Red AEC Substrate Chromagen Kit or equivalent---Kit
9. Mayer's Hematoxylin (commercial)

All reagents are applied at 200-250ul per slide.

Uncontrolled

SUPPLIES

Calibrated pipettes
Pipette tips
Tubes
Incubating Waterbath
Calibrated scale
Slide straining racks
Coverplates

SPECIAL SAFETY PRECAUTIONS

Appropriate laboratory wear i.e.: gloves, lab coat/apron, and protective eyewear

EQUIPMENT CALIBRATION AND MAINTENANCE

N/A

QUALITY CONTROL

Positive and negative controls are used to validate the results of this test.

TEST METHOD INSTRUCTIONS

1. Cut 4-5 micron sections and mount on charged slides. Allow slides to dry overnight.
2. Bake slides in a 60°C oven for 30 minutes(*this step can also be done on the autostainer).
3. *Deparaffinize and hydrate slides according to standard protocol.
4. Quench in endogenous peroxidase solution for 10 mins.
5. Rinse slide in running deionized water for 10 minutes.
6. Place slides in pre-heated antigen retrieval solution in a 37°C water bath for 15 minutes.
7. Rinse slides in running deionized water for 10 minutes.
8. Put slides in staining rack.
9. Rinse slides in rinse buffer for 10 minutes.
10. Apply Block and incubate for 10 minutes.
11. **WITHOUT RINSING** apply primary antibody to positive control slide and one test tissue slide. Apply I-1000 to negative control slide and one test tissue slide then incubate 90 to 120 minutes.
12. Rinse slides in rinse buffer for 10 minutes.
13. Apply Dako Envision Rabbit HRP to all slides and incubate for 30 minutes.
14. Rinse slides in rinse buffer for 10 minutes.
15. Apply Nova Red chromogen to all slides and incubate for 5 to 10 minutes at room temperature.
16. Remove slides from immuno staining rack and put into regular staining rack.
17. Rinse slides in distilled water for 5 minutes.
18. Counterstain in Mayer's Hematoxylin for 5 to 10 minutes.
19. **Wash in running tap water for 5 minutes.
20. Dehydrate and clear.
21. Coverslip.

*Baking, deparaffinization and rehydration steps can be done on the autostainer using program "Xylene to H2O w/oven".
**Can be washed, dehydrated and cleared on the autostainer using program "immuno finish".

CALCULATIONS

N/A

EXPECTED VALUES

N/A

Uncontrolled

INTERPRETATION OF RESULTS

1. Specific stain-red to brownish red with Nova Red
2. Non-specific stain-no color to light pink
3. Nuclei-blue

METHOD LIMITATIONS

N/A

POST ANALYTICAL PROCEDURES

1. Enter case into Antigen Detection QC Log.
2. Enter workload into StarLims to release.

VALIDATION

N/A

REFERENCES AND RELATED DOCUMENTATION

N/A

APPENDICES

1. Suggested Reagent Source and Use
    a. Antigen Retrievel Solution
        i. Pepsin
            a. Sigma, catalog # P7000

    b. Specific Primary Antibody
        i. anti-C. perfringens - rabbit, polyclonal
            a. GenWay Biotech, Inc catalog # 18-783-77463

    c. Nonspecific Primary Antibody
        i. Rabbit IgG
            a. Vector Labs, catalog #I-1000, use same dilution as primary antibody

    d. Secondary Detection Solution
        i. Horseradish Peroxidase Labeled Polymer-anti-rabbit
            a. DakCytomation, catalog #K4001, ready to use

    e. Chromagen Substrate Solution
        i. Nova Red
            a. Vector Labs, catalog #SK-4800, follow kit instructions

Uncontrolled
