# Supplementary material for: Gut microbiota and age shape susceptibility to clostridial enteritis in lorikeets under human care
Source: Anim Microbiome. 2022 Jan 9;4:7. doi: 10.1186/s42523-021-00148-7 (PMC8744333; doi:10.1186/s42523-021-00148-7)
Supplement: Supplementary file 3 — Additional file 3. Reagent preparation for measurement of Trypsin Inhibitor Levels in nectar. [file 42523_2021_148_MOESM3_ESM.docx]

**Additional File 3: Measurement of Trypsin Inhibitor Levels in Nectar**

**Methods**

*Reagent preparation*

A stock solution of Tris buffer was made by adding 6.05 g Tris (Sigma-Aldrich, St. Louis, MO) with 2.94 g calcium chloride (CaCl_2_) (Sigma-Aldrich, St. Louis, MO) to approximately 20 mL deionized water. The pH of the solution was adjusted to 8.2 with 1N hydrochloric acid (Thermo Fisher Scientific, Waltham, MA), then the solution was brought up to a total volume of 50 mL with water. To make the working solution, 5 mL Tris stock solution was combined with deionized water to a final volume of 100 mL (1:20 dilution). A trypsin stock solution was produced by adding 20 mg bovine trypsin (Sigma-Aldrich, St. Louis, MO) to 50 mL 0.001M HCl. To make the working solution, a 1:20 dilution was performed to yield a concentration of 0.02 mg trypsin/mL. The substrate solution was prepared by mixing 40 mg benzoyl-DL-arginine-p-nitroanilide hydrochloride (BAPA) (Sigma-Aldrich, St. Louis, MO) with 1 mL dimethyl sulfoxide (Sigma-Aldrich, St. Louis, MO). The mixture was gently heated on a hot plate at 32◦C to aid with dissolution before being added to 100 mL Tris buffer. This solution was incubated in a Precision SWB 15, Model TSSWB15 water bath (Thermo Scientific, Waltham, MA) set at 37◦C. A 30% acetic acid solution was made by combining 30 mL glacial acetic acid (Thermo Fisher Scientific, Waltham, MA) with 70 mL deionized water.
