# Supplementary figures and images for: Gut microbiota and age shape susceptibility to clostridial enteritis in lorikeets under human care
Source: Anim Microbiome. 2022 Jan 9;4:7. doi: 10.1186/s42523-021-00148-7 (PMC8744333; doi:10.1186/s42523-021-00148-7)

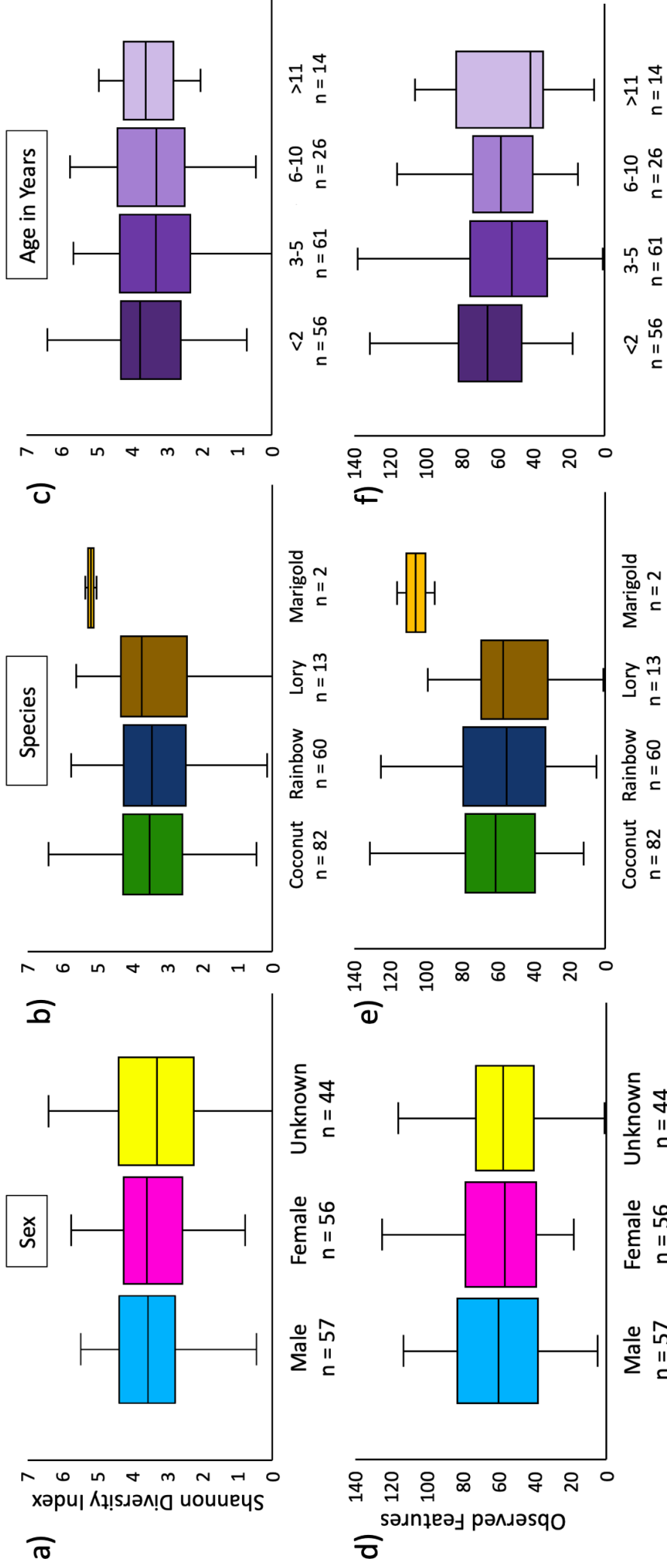

Supplement: Supplementary file 4 — Additional file 4. Microbial community diversity in healthy CZA birds by sex, species, and age. Healthy birds across all time points: Microbial diversity (Shannon Diversity Index and Observed Features) did not differ significantly by a,d) sex, b,e) species, or c,f) age (Kruskal Wallis: Shannon sex p = 0.77, species p = 0.25, age p = 0.89; Observed Features sex p = 0.93, species p = 0.18, age p = 0.08). [file 42523_2021_148_MOESM4_ESM.pdf]

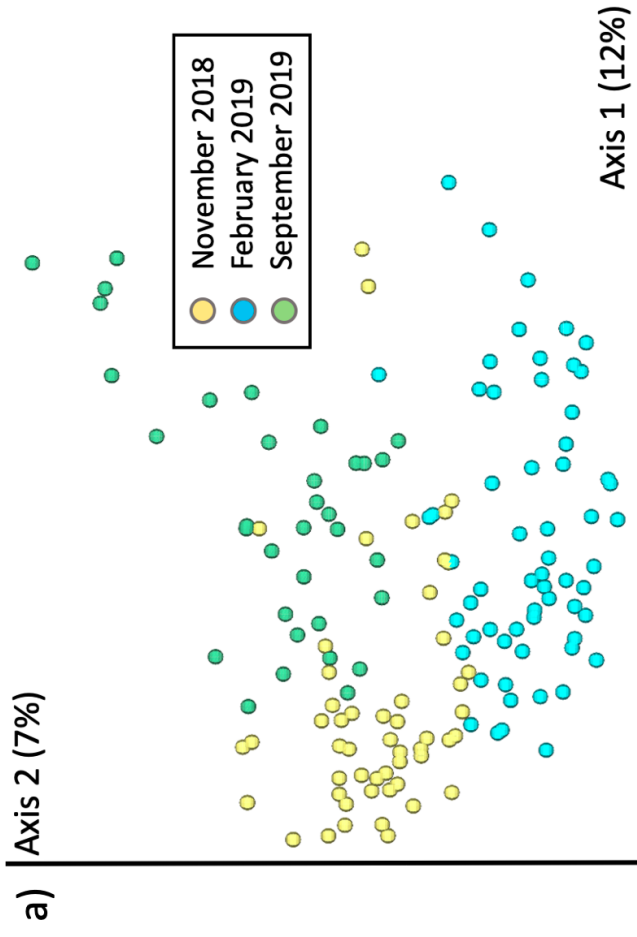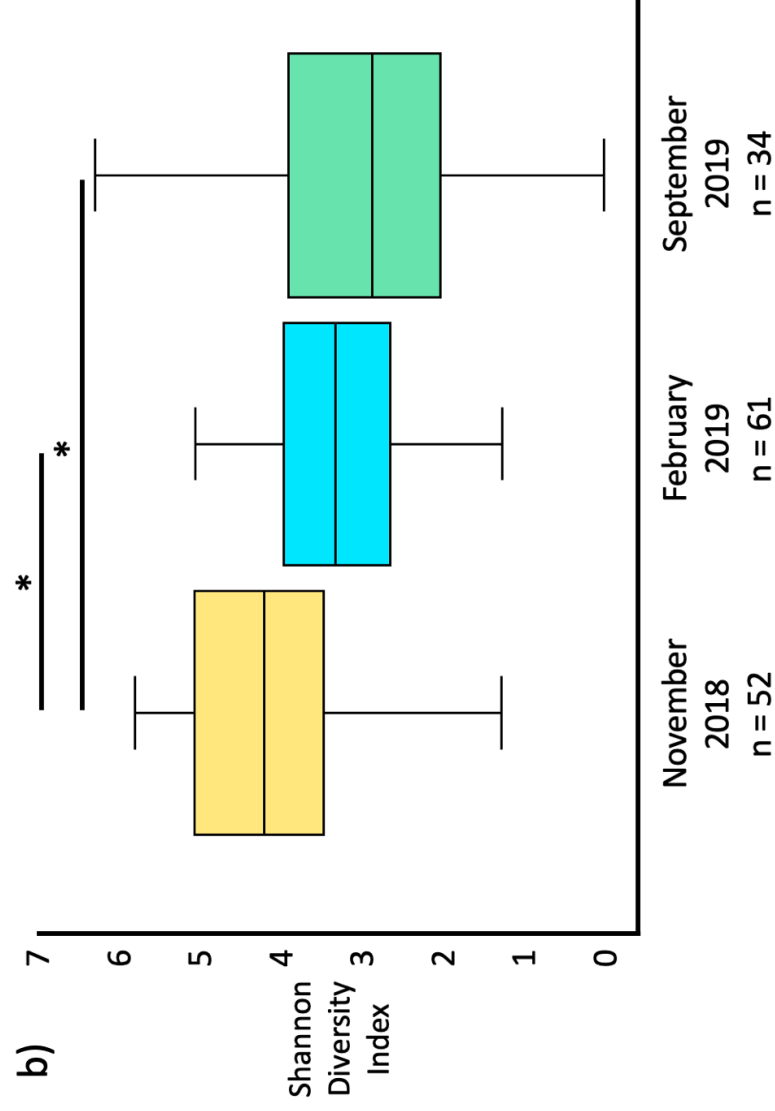

Supplement: Supplementary file 6 — Additional file 6. Seasonality in gut microbiota in healthy CZA lorikeets. a) Microbial community composition (Unweighted UniFrac) and b) diversity (Shannon Diversity Index) by season (November 2018, February 2019, September 2019). There were significant shifts in microbial composition by season (PERMANOVA p = 0.001), and microbial diversity decreased significantly between November 2018 and September 2019 (Kruskal-Wallis *p < 0.001). (Also see Fig. 1.) [file 42523_2021_148_MOESM6_ESM.pdf]

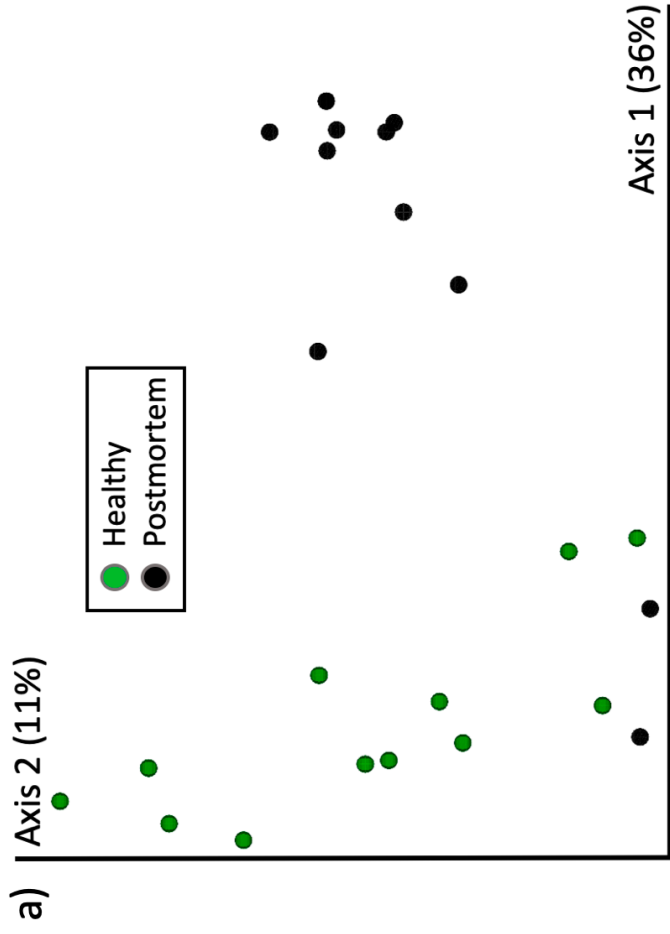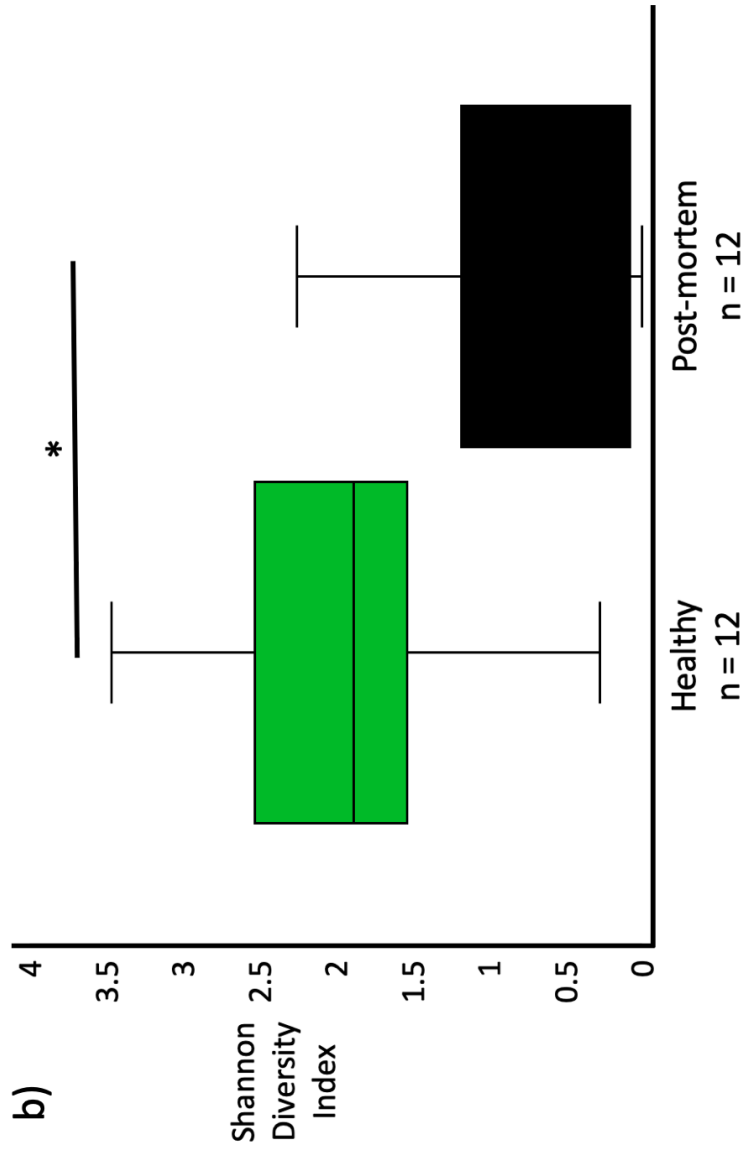

Supplement: Supplementary file 11 — Additional file 11. Altered microbial composition and diversity in Denver Zoo lorikeets with enteritis. Microbial composition and diversity in healthy lorikeets and lorikeets that died or were euthanized due to enteritis (post-mortem). a) Microbial composition (Unweighted UniFrac) was significantly altered (PERMANOVA p = 0.001) and b) microbial diversity was significantly decreased (Shannon, Kruskal-Wallis *p < 0.005) in post-mortem lorikeets. (Also see Fig. 4.) [file 42523_2021_148_MOESM11_ESM.pdf]

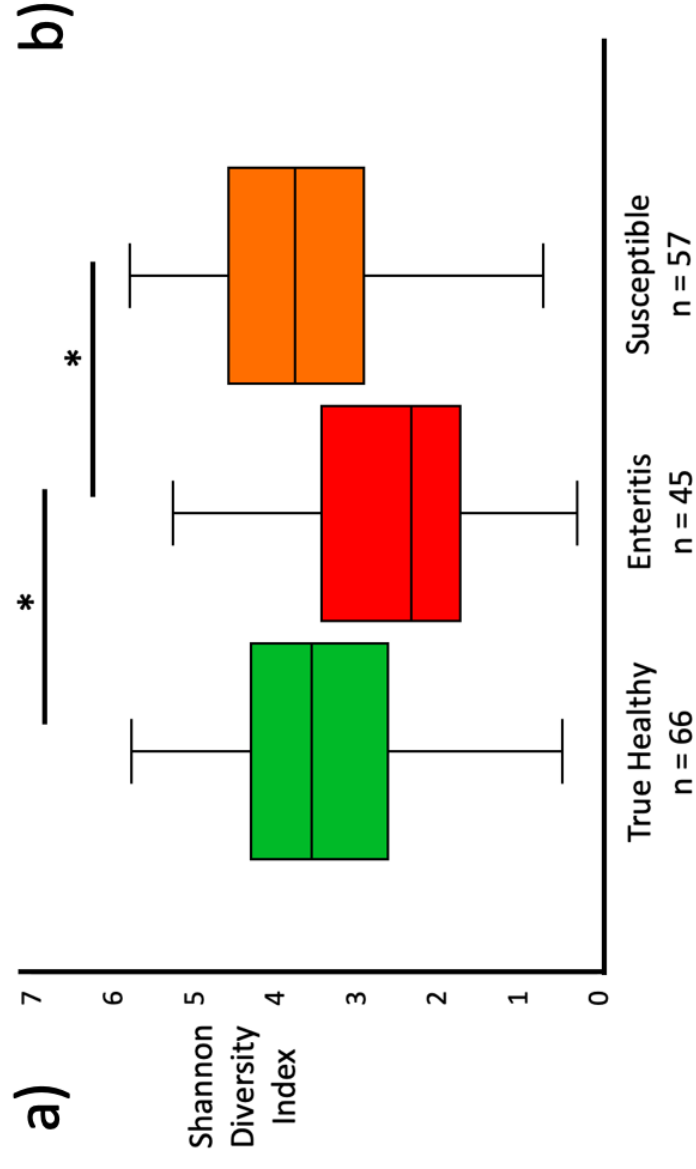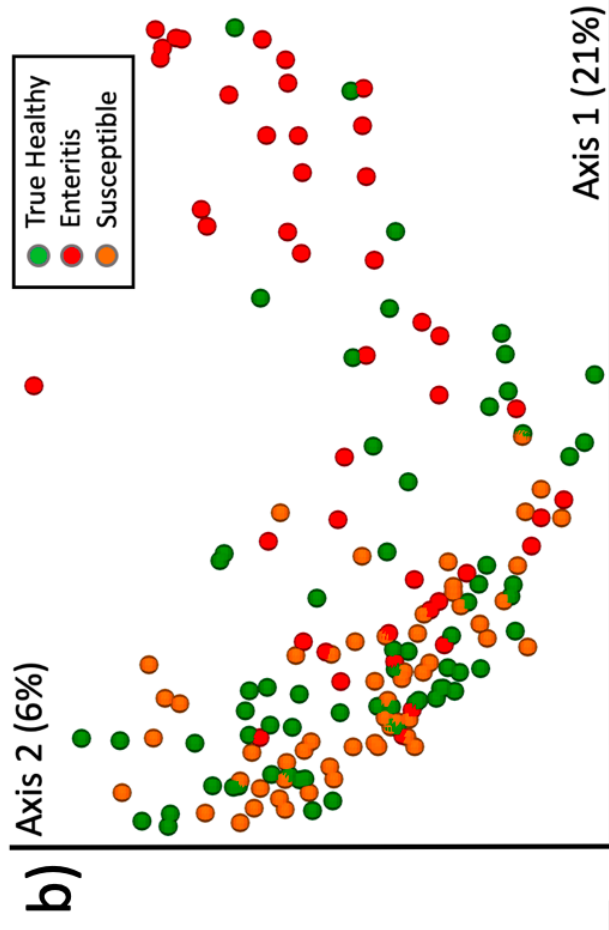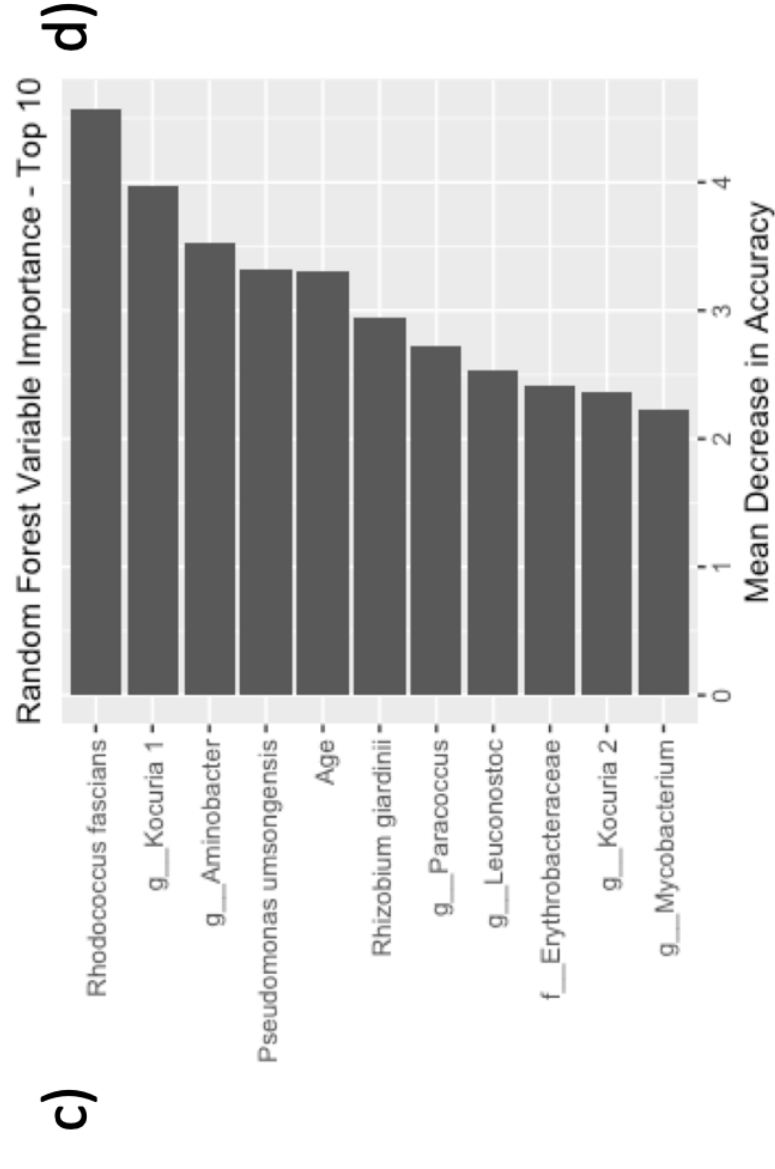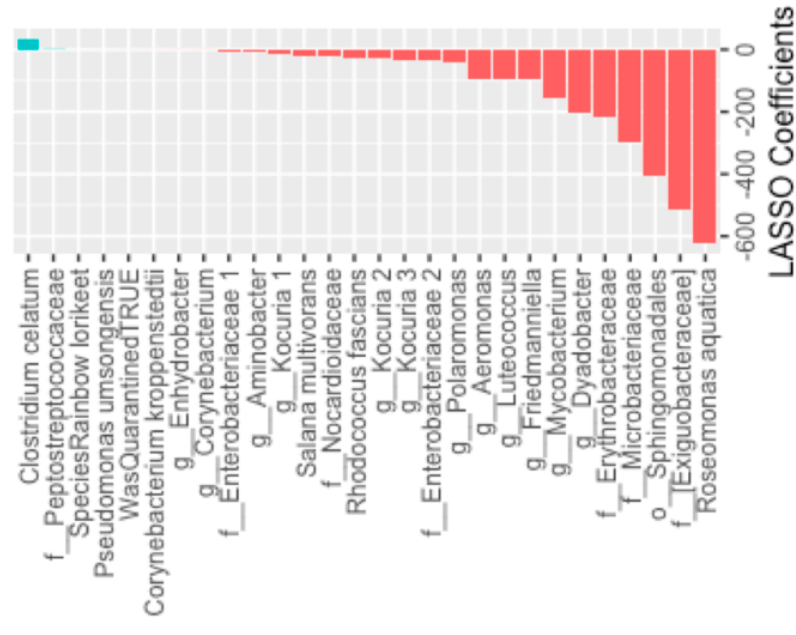

Supplement: Supplementary file 12 — Additional file 12. Susceptible CZA lorikeets have altered microbial composition that predicts enteritis. Healthy lorikeets that never developed enteritis throughout the sampling period were identified as “True Healthy” while healthy birds that developed enteritis at least once during the sampling period were identified as “Susceptible.” “Enteritis” represents birds with enteritis that were sampled while they were clinically ill. No post-mortem samples are included in this figure. a) Microbial diversity (Shannon, Kruskal-Wallis, p < 0.005) was increased in Susceptible and True Healthy birds as compared to birds with enteritis and b) microbial composition (Unweighted UniFrac) was altered in Susceptible birds. Variables associated with susceptibility or health were then predicted by a c) Random Forest (RF) or d) LASSO model. The RF model has a sensitivity of 0.75, a specificity of 0.571, and an overall accuracy of 0.557. This model identifies the relative importance of each variable but not whether the variable is associated with susceptibility or health. The LASSO model has a sensitivity of 0.875, a specificity of 0.571, an overall accuracy of 0.733, and generates an area under the curve (AUC) of 0.72. This model (LASSO) identifies whether a variable is associated with susceptibility or health but not the relative importance of the variable. [file 42523_2021_148_MOESM12_ESM.pdf]
