# Supplementary material for: Gut microbiota and age shape susceptibility to clostridial enteritis in lorikeets under human care
Source: Anim Microbiome. 2022 Jan 9;4:7. doi: 10.1186/s42523-021-00148-7 (PMC8744333; doi:10.1186/s42523-021-00148-7)
Supplement: Supplementary file 7 — Additional file 7. Altered microbial diversity and composition in CZA lorikeets with enteritis. Microbial composition and diversity in healthy lorikeets, lorikeets with enteritis, and lorikeets that died or were euthanized due to enteritis (post-mortem). a) Microbial composition (Unweighted UniFrac) was significantly altered (PERMANOVA p = 0.001) and b) microbial diversity (Shannon Diversity Index) was significantly decreased (Kruskal-Wallis *p < 0.005, **p < 0.0005, ***p < 0.00001) in lorikeets with enteritis or post-mortem lorikeets. c) Taxa bar plots showing taxonomic distributions within healthy lorikeets, lorikeets with enteritis, and post-mortem lorikeets. Clostridium perfringens is denoted in orange and is found at signiciantly increased abundances in enteritis and post-mortem samples. (Also see Fig. 2.) [file 42523_2021_148_MOESM7_ESM.pdf]

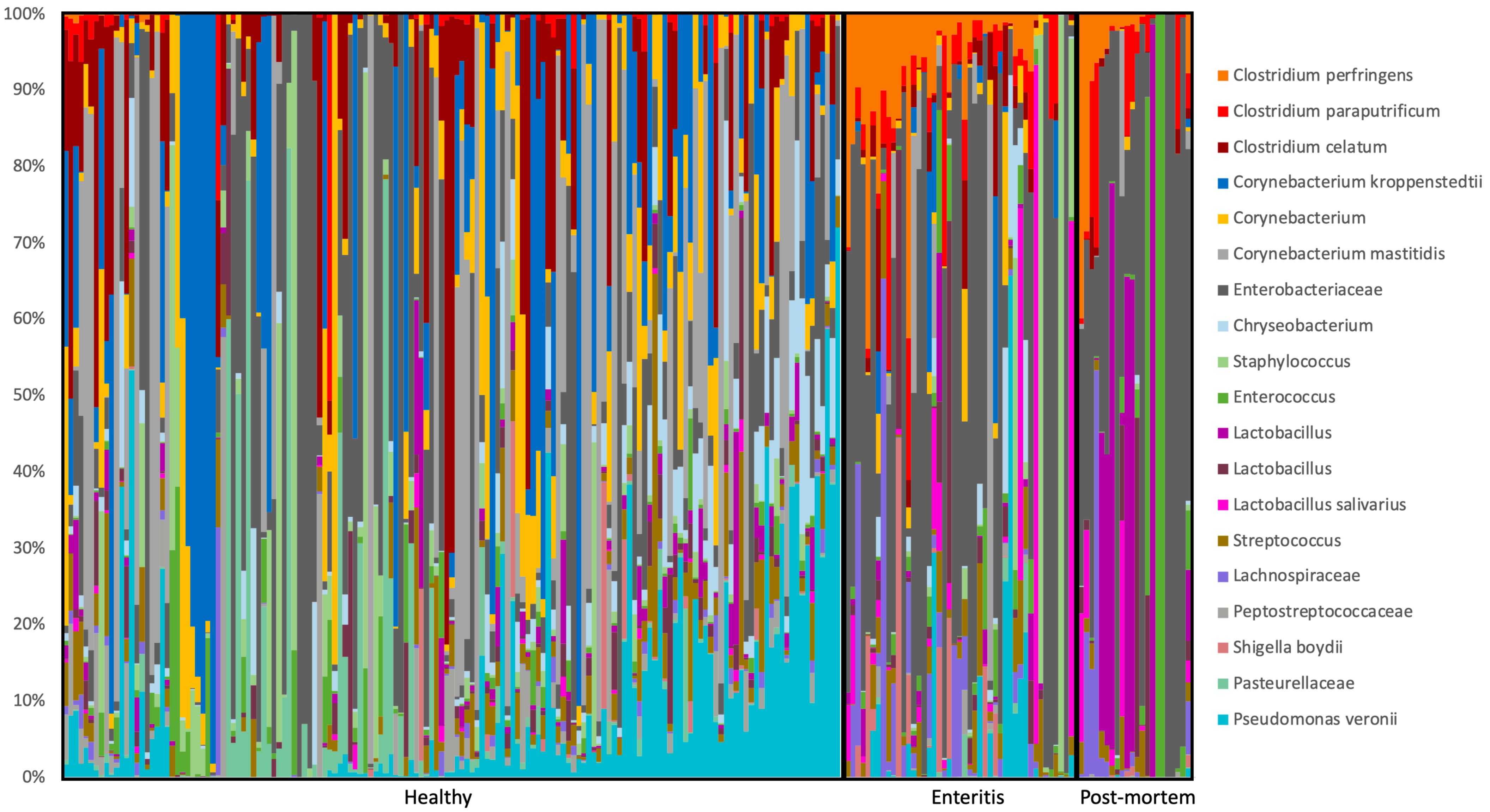
100%
90%
80%
70%
60%
50%
40%
30%
20%
10%
0%
Healthy
Enteritis
Post-mortem
Clostridium perfringens
Clostridium paraputrificum
Clostridium celatum
Corynebacterium kroppenstedtii
Corynebacterium
Corynebacterium mastitidis
Enterobacteriaceae
Chryseobacterium
Staphylococcus
Enterococcus
Lactobacillus
Lactobacillus
Lactobacillus salivarius
Streptococcus
Lachnospiraceae
Peptostreptococcaceae
Shigella boydii
Pasteurellaceae
Pseudomonas veronii
